# Complete Genome Sequencing and Transcriptome Analysis of Nitrogen Metabolism of *Succinivibrio dextrinosolvens* Strain Z6 Isolated From Dairy Cow Rumen

**DOI:** 10.3389/fmicb.2020.01826

**Published:** 2020-08-14

**Authors:** Samson Hailemariam, Shengguo Zhao, Jiaqi Wang

**Affiliations:** State Key Laboratory of Animal Nutrition, Institute of Animal Sciences, Chinese Academy of Agricultural Sciences, Beijing, China

**Keywords:** *Succinivibrio dextrinosolvens*, nitrogen metabolism, genome sequence, enzyme activity, rumen

## Abstract

The unclassified *Succinivibrionaceae* lineages are abundant in high yielding multiparous cows, and their presence is positively correlated with milk yield and fat percentage and reduces methane emissions. However, it is still unclear which species are associated with the most efficient feed nutrient utilization and productivity. Here, we used integrated whole genome sequencing and matrix-assisted laser desorption/ionization mass spectrometry, coupled with phenotypic and chemotaxonomic analysis, to characterize *S. dextrinosolvens* Z6, a species in *Succinivibrionaceae* isolated from the rumen. To assess the role of *S. dextrinosolvens* Z6 in nitrogen metabolism, cells grown in different nitrogen sources were analyzed by RNA sequencing. The whole genome sequence result revealed a genome size of 3.47 Mbp with 38.9% of G + C content. A total of 2993 encoding sequences account for 98%. The genes for regulating carbohydrate (10.6%) and amino acid (9%) transport and metabolism were the most abundant. ANI (Average nucleotide identity) showed that SD-Z6 was most closely related to SD-22B (99.96%). The whole genome alignment of SD-Z6 with SD-22B showed a more than 0.34 Mb nucleotide difference. Growth of SD-Z6 occurred at a temperature 36–42°C with an optimum at 39.7°C, pH 6–8; the optimum pH was 6.9 and with 0–1% (w/v) NaCl. The maximum growth (OD_600_ 0.825 ± 0.12) and microbial crude protein (MCP) (178.2 μg/ml) were observed in cells grown in amino acid. The maximum concentration of ammonia (3.96 ± 1.2) was observed in urea containing media and 1.06 mM (26.7% of the produced) remained after 24 h incubation. Activities of urease and glutamine synthase (*P* < 0.01) and glutamate dehydrogenase (*P* < 0.05) were significantly different in nitrogen and growth phase. Glutamate synthetase (*P* < 0.01) was significantly different only at different growth phases. In total, 1246 differentially expressed genes (DEGs) were identified in all nitrogen. Among DEGs, 33 were related to nitrogen metabolism. Their expression correlated with nitrogen sources and the intensity of enzyme activity. This result enhances our understanding of the roles of *Succinivibrionaceae* in the efficient nitrogen utilization and on environmental protection.

## Introduction

The rumen is a complex ecosystem in which consumed nutrients are digested and converted to diverse end products by microorganisms. The main end products of fermentation are volatile fatty acids (VFAs) and microbial protein, both of which are used by the host ruminant as sources of energy and protein and are important to the health and productivity of the animals (Bryant, 1959; Russell and Hespell, 1981). However, the efficiency of VFA and microbial protein production by the fermentation in the rumen is highly variable and dependent on genetic and functional aspects of the rumen microbiome (Khafipour et al., 2009; Kruger Ben Shabat et al., 2016; Zeng et al., 2019). The unclassified *Succinivibrionaceae* is among different ruminal bacteria families, whose presence in the rumen has been associated with feed efficiency and animal productivity for dairy cattle fed with high total mixed ration (TMR) (Hernandez-Sanabria et al., 2012; Indugu et al., 2017; Zeng et al., 2019).

Studies by Indugu et al. (2017) and Xue et al. (2018) revealed that *Succinivibrionaceae* lineages were abundant in high yielding primiparous and multiparous cows and that their presence was positively correlated with milk yield and milk fat composition. Groups of bacteria in this family have been reported to produce succinate, a precursor of propionate (Blackburn and Hungate, 1963) and acetate, which affect lactation performance and milk fat content, respectively. Moreover, Pope et al. (2011), McCabe et al. (2015), and Danielsson et al. (2017) reported that unclassified *Succinivibrionaceae* growth in the rumen reduces methane emissions since bacteria in this family utilize hydrogen to produce succinate, which is also an input for methanogenesis. However, these studies did not target specific *Succinivibrionaceae* species and the main focus was on the contribution of the bacteria in this family to carbohydrate metabolism in the rumen. Notably, their contribution to nitrogen metabolism has not been studied in detail, despite the fact that the balance of carbon and nitrogen utilization is a key factor in the growth and performance of ruminants (Seo et al., 2013).

The *Succinivibrionaceae* family consists of gammaproteobacteria, which are gram-negative, strictly anaerobic, non-spore-forming species, and comprises mainly ruminant inhabitant members of the *Succinivibrio*, *Succinimonas*, and *Ruminobacter* genera. *Succinivibrio dextrinosolvens* is a well-studied species of the *Succinivibrionaceae* family and *Succinivibrio* genus (Bryant and Small, 1955). Previously isolated *S. dextrinosolvens* cells were shown to be motile in vibrating movements and to appear singly or in pairs; however, some isolated strains commonly form helical or twisted filaments of two to four cell coils (Stackebrandt and Hespell, 2006). *Succinivibrio* strains ferment glucose, resulting in the production of large amounts of acetic and succinic acids (Bryant and Small, 1955). Moreover, a few strains have been shown to possess all the enzymes necessary for nitrogen-containing compound degradation and assimilation. For example, *S. dextrinosolvens* C18 has glutamine synthetase (GS), urease, glutamate dehydrogenase, and several other nitrogen assimilation enzymes, whereas strain 24 lack urease (Patterson and Hespell, 1985). Such differences between *S. dextrinosolvens* strains could be important for their ability to participate in nitrogen metabolism. However, the absence of complete genome information for this bacterium has been a major impediment to functional gene studies, and the different descriptions of *S. dextrinosolvens* strains have mainly been based on morphological, biochemical (Bryant and Small, 1955; O’Herrin and Kenealy, 1993), or partial genomic characteristics. In addition, little is known about the transcriptional response of this bacterium to various nitrogen sources. We report the genomic, biochemical, molecular, and transcriptome features of *S. dextrinosolvens* Z6 and propose that this information will benefit the manipulation of its genetic potential for better feed utilization and the production performance of ruminant animals.

## Materials and Methods

### Media Components and Preparation

The basal media contained 30 ml of clarified rumen fluid, 10 ml of urea (20% w/v), 0.05 g of glucose, 0.05 g of maltose, 0.05 g of soluble starch, 38 ml of solution 1 (0.3% K_2_HPO_4_), 38 ml of solution 2 (0.3% KH_2_PO_4_, 0.6% NaC1, 0.06% MgSO_2_, and 0.06% CaCl_2_), 0.1 ml of Pfennig trace elements (300 mg of H_3_BO_3_, 100 mg of ZnSO_4_⋅7H_2_O, 30 mg of MnCl_2_⋅4H_2_O, 20 mg of CoCl_2_⋅6H_2_O, 30 mg of Na_2_MoO_4_⋅2H_2_O, 10 mg of Na_2_SeO_3_, 20 mg of NiCl_2_, 10 mg of CuCl_2_⋅2H_2_O, and 150 mg of FeCl_2_⋅4H_2_O per 1000 ml of distilled H_2_O), 5 ml of hemin (0.05%), 0.31 ml of VFA mix (17 ml of acetic acid, 6 ml of propionic, 4 ml of *n*-butyric, and 1 ml each of *n*-valeric, isovaleric, isobutyric, and 2-methylbutyric acids), 0.5 g of NaHCO_3_, 0.1 ml of resazurin (0.1%), 0.05 g of L-cysteine HCl, and 0.0012 g of phenol red per 100 ml. Media one (M1) consisted of basal media plus 0.05 g of tryptone and 0.05 g of yeast extract, while basal media alone was used as media two (M2), media three (M3) was basal media and 1.5 g agar per 100 ml, and media four (M4) was basal media without urea. After boiling, a gassing probe was used to purge the solution with food-grade CO_2_ for at least 2 h while the media cooled. Subsequently, 10 ml of media was dispensed anaerobically into a Hungate tube in an anaerobic chamber, and autoclaved for 15 min at 100 kPa, 121°C. After cooling, urea, vitamins, and phenol red were added (as above) and the media was stored at 4°C in the dark.

### Enrichment for and Isolation of *S. dextrinosolvens*

*S. dextrinosolvens* Z6 was isolated from rumen fluid obtained from dairy cows fed with basic TMR as previously described (Jin et al., 2016). To prepare the rumen microbe inoculum, the rumen fluid sample was aspirated with a syringe and added to an anaerobically prepared 30% (v/v) glycerol stock solution in equal volumes. The inoculum samples were stored on dry ice and immediately brought back to the laboratory and stored at −80°C.

For the enrichment, 200 μl of rumen microbe inoculum was inoculated by using a syringe inside the anaerobic chamber into 10 ml of M1 media and cultured at 39°C for 24 h as the first generation. The culture was then transferred to new M1 media to produce the second and third generations. The third-generation culture was diluted and spread on M3 media and incubated at 39°C for 2–4 days in an incubator inside an anaerobic controlled atmosphere chamber (Lansing, MI, United States). Pink colonies were picked and cultured in M2 media at 39°C for 16 h for genomic DNA extraction using the TIANamp Bacteria DNA Kit (TIANGEN, China). In parallel, the same amount of bacterial liquid solution was mixed with 30% (v/v) glycerol cryopreservation solution, and the strain was stored at -80°C.

PCR primers [UreC-F (5’-TGGGCCTTAAAATHCAYGA RGAYTGGG-3’) and UreC-R (5’-SGGTGGTGGCACACCAT NANCATRTC-3)] that amplify the urease alpha subunit encoding gene (*ureC*) were used to determine the number of *ureC* gene copies (Reed, 2001). The 16S rRNA gene from total bacteria was quantified using the universal primers 27F (5′-AGAGTTTGATCMTGGCTCAG-3′) and 1492R (5′-TACGGYTACCTTGTTACGACTT-3′) (Frank et al., 2008). All PCR reactions were performed with a MyCycler Thermal Cycler (Bio-Rad, United States) in a 25 μl mixture containing 2.5 μl of PCR buffer (Invitrogen), 0.75 μl of MgCl_2_ (50 mM), 0.5 μl of dNTP (10 mM), 1.5 μl of each forward and reverse primer, 0.3 μl of platinum Taq DNA polymerase (Invitrogen), 1 μl of template DNA, and 16.95 μl of ddH_2_O. PCR amplification began with a 3 min denaturing step at 94°C, followed by 30 cycles at 94°C for 30 s, 58°C for 30 s, and 72°C for 1.5 min, and the final extension was at 72°C for 7 min and 10°C for 10 min for 16S rRNA amplification. For the urease amplification, cycling conditions were as follows: 5 min 94°C and 30 cycles of 94°C for 30 s, 50°C for 30 s, and 72°C for 30 s, with a final extension at 72°C for 15 min and 10°C 10 min. The PCR products were sequenced by Sanger sequencing (Applied Biosystems 3730XL, Foster City, CA, United States).

For urease activity testing, cell-free extracts were used as crude enzymes in an experiment modified from previous methods (Richmond and Yep, 2019). Briefly, 2 ml of each anaerobic culture was harvested by centrifugation at 12,000 *g* for 20 min at 4°C, washed once with 50 mM PBS buffer, and resuspended in PBS buffer. The cells were disrupted by sonication at 80 W (30 cycles of a 30 s pulse followed by 1 min of cooling in an ice water bath). After centrifugation (12,000 *g*, 20 min, 4°C), the supernatant was collected as the enzyme solution for urease activity measurement.

### Whole Genome Sequencing and Analysis

Genomic DNA from *S. dextrinosolvens* Z6 isolated as above was sequenced using Oxford Nanopore Technology (Oxford, United Kingdom). For ONT sequencing, high-quality genomic DNA was extracted using the QIAamp genomic DNA kit (Qiagen, California, United States) following the manufacturer’s instructions. DNA quality was confirmed using gel electrophoresis and a NanoDrop One (Thermo Fisher Scientific, CA, United States), and the DNA was quantified using a Qubit (Thermo Fisher Scientific, CA, United States). The library was prepared using the ONT 1D ligation sequencing kit (Oxford, United Kingdom), and the final library was loaded onto a flow cell and sequenced on a GridION Sequencer device (Oxford, United Kingdom) performing single-molecule DNA sequencing to obtain raw sequence data.

The following criteria were used as a cutoff in a quality control of the raw data: mean *Q* score template ≥ 7 and sequence length ≥1000 bp. After quality control, the data were assembled using canu (Koren et al., 2017) and then corrected by Pilon (Walker et al., 2014). After removing redundant sequences, circlator (Hunt et al., 2015) was implemented to move the origin of the sequence to the start site of the genome replication to obtain the final genome sequence. The data were aligned to the assembled genome using minimap2 (Li, 2018), and the sequence depth of each locus was calculated by SAMtools (Li et al., 2009). Plasmid sequence was identified by aligning the sequences to a plasmid database (Jesus et al., 2019) using blastn software (Camacho et al., 2009), and coding genes were predicted by prodigal (Hyatt et al., 2010). The tRNA genes were predicted by tRNAscan-SE (Lowe and Chan, 2016), rRNA genes were predicted by RNAmmer (Lagesen et al., 2007), and other ncRNAs were predicted by infernal (Nawrocki and Eddy, 2013) searching the Rfam database (Kalvari et al., 2018). CRISPR was predicted by minced (Bland et al., 2007), and Islander (Hudson et al., 2015) was used for predicting gene islands. Interproscan (Jones et al., 2014) was used to annotate the predicted genes from the TIGRFAMs (Haft et al., 2013), Pfam (Kalvari et al., 2018), and GO (Ashburner et al., 2000) databases. Blastp was used to align the protein sequences to the Kyoto Encyclopedia of Genes and Genomes (KEGG) (Kanehisa et al., 2014) and RefSeq databases (O’Leary et al., 2016). The protein sequences were also aligned to the clusters of orthologous groups (COG) database using rpsblast (Camacho et al., 2009) for COG annotation.

For genome comparison between *S. dextrinosolvens* Z6 and other related members of the genus, draft genome sequences of four *S. dextrinosolvens* strains and one *Succinimonas amylolytica* strain were downloaded from the NCBI database (accession numbers: GCA_900167015.1, GCA_900116345.1, GCA_000702045.1, GCA_900114195.1, and NZ_KB899636.1 for strain DSM 3072, ACV-10, H5, 22B, and *S. amylolytica* DSM_2873 used as an outgroup). Circular comparison plots between these genomes were prepared using BLAST Ring Image Generator (BRIG) (version 0.95) (Alikhan et al., 2011), and inference of clusters of orthologous groups was performed with OrthoFinder (version 1.1.5) (Emms and Kelly, 2015). A total of 1044 gene families exclusively containing single copy genes were selected as potential phylogenetic markers. For each of these clusters of orthologous groups, protein family alignments were built with muscle (version 3.8) (Edgar, 2004). Phylogenetic trees were constructed with RAxML (version 8.2.9 SSE3) (Stamatakis, 2014). To calculate the average nucleotide identity (ANI) between strains, the OAT (Orthologous ANI Tool) software package was used (Lee et al., 2016). Venn maps were made using the VennDiagram R package (Chen and Boutros, 2011).

### Phenotypic Characterization

Cell morphology was examined using scanning electron microscopy (Hitachi FE-SEM SU-8010). Gram staining was performed using Hucker’s modification (Hucker, 1921). The growth of *S. dextrinosolvens* Z6 was analyzed at different temperatures (15, 20, 25, 30, 37, 40, and 45°C) under anaerobic conditions at 39°C at different pH values (pH 5, 6, 7, 8, 9, and 10), and in different NaCl concentrations [0, 0.5, 1, 2, 3, 4, and 5% (w/v)] to test salt tolerance. M2 media without carbohydrate sources was used to test carbohydrate (glycerol, *D*-cellobiose, *D*-mannose, *D*-raffinose, *D*-sorbitol, *L*-rhamnose, *D*-trehalose, *D*-glucose, *D*-mannitol, *D*-lactose, *D*-saccharose, *D*-maltose, salicin, *D*-xylose, starch, and *L*-arabinose) utilization. Each carbohydrate was added to the carbohydrate free medium to a 0.5% final concentration and the media was prepared in triplicate and filter sterilized, before fresh overnight cultures were inoculated and incubated for 72 h at 37°C. The utilization of carbohydrate was determined by OD_600_ readings as previously described by Bertin et al. (2013), and the activities of 20 enzymes were determined using the API ZYM system (BioMerieux, Marcy, France) according to the manufacturer’s instructions.

### Nitrogen Metabolism

*S. dextrinosolvens* Z6 cells were grown anaerobically at 39°C in M4 media supplemented with different nitrogen sources [ammonia ((NH_4_)_2_SO_4_), Amicase amino acid mix (Sigma-Aldrich, United States), and urea] in a concentration of 9.4 mM NH_4_-N. Five independent biological replicates were analyzed for each treatment. Growth was measured in five replicates by determining the increase in optical density at 600 nm (OD_600_) using a Visible Spectrophotometer V-5600PC (Shanghai Metash Instruments Co., Ltd., Shanghai, China) for 72 h. The maximum growth rate of the bacteria was determined as in Hall et al. (2014). Samples for chemical and enzymatic assays were harvested at early, mid, and late log growth phases by centrifugation at 8350 *g* for 5 min. The cell pellets and supernatants were stored at −80°C until analysis. The transcriptome samples were harvested in the mid-exponential growth phase.

### Chemical and Enzyme Activity Analysis

Ammonia concentration was determined in the supernatant using an automated phenol-hypochlorite method (Broderick and Kang, 1980). The concentration of urea was determined using a urea assay kit (Sigma-Aldrich) based on the manufacturer’s protocol. The concentration of total free α-amino acids in the supernatant of the mixed amino acid-grown cultures was analyzed using ninhydrin as previously described (Jones et al., 2002). Microbial crude protein (MCP) was determined by the Folin phenol method (Makkar et al., 1982). Urease and glutamine synthase (GS) activity were determined using urease and glutamine synthase assay kits (Sigma-Aldrich), respectively, and glutamate dehydrogenase (GDH) and glutamate synthetase (GOGAT) activity were measured using activity detection kits (Solarbio, Beijing, China) according to the manufacturer’s instructions.

### Transcriptomic Analysis

*S. dextrinosolvens* Z6 was grown in three different nitrogen sources in five biological replications and then harvested in the mid-exponential growth phase by centrifugation at 10,000 *g* for 5 min at 4°C to stop further growth of the bacteria. Total RNA was extracted from bacterial cell samples using TRIzol (Invitrogen, CA, United States) and purified using a QIAGEN RNeasy Mini Kit (Qiagen) following the manufacturer’s instructions. The concentration and purity of the extracted RNA were detected on a NanoDrop2000 Spectrophotometer (Thermo Fisher Scientific, MA, United States), and RNA integrity was visualized by agarose gel electrophoresis and on an Agilent 2100 Bioanalyzer (Agilent Technologies, CA, United States). Ribosomal RNA (rRNA) was removed from total RNA by use of a Ribo-off rRNA Depletion Kit (Vazyme Biotech, Nanjing, China), and the libraries were constructed using the TruSeq Stranded Total RNA Library Prep Kit (Vazyme Biotech, Nanjing, China). The libraries were then sequenced using the Illumina HiSeq platform for 2 × 150 bp sequencing. RNA-seq quality control of raw data was performed using the FastQC^1^ and HTQC tools (Yang et al., 2013). Once the samples had passed the quality assessment, depending on the quality assessment results, the RNA-seq read quality trimming was performed using the sickle software (Version1.33) (Fass, 2011) and SeqPrep^2^ program. The high-quality sequences were aligned to the complete genome of *S. dextrinosolvens* Z6 using Burrow-Wheeler Aligner (BWA) and DIAMOND (Buchfink et al., 2014). High-throughput functional annotation was performed using Blast2GO (Version 2.5). The reference genome was analyzed using six major databases (NR, Swiss-Prot, Pfam, COG, GO, and KEGG) to obtain gene function information. The quantitative expression software RSEM (Li and Dewey, 2011) was used to quantitatively analyze the expression levels of genes and transcripts.

Differentially expressed genes (DEGs) between different nitrogen source groups were determined using the R/Bioconductor package, DESeq (Anders and Huber, 2010) with read counts calculated by HTseq (Anders et al., 2015). Read count normalization was performed using the regularized logarithm (rlog) method in DESeq. DEGs were selected if the adjusted *p* < 0.05 and the fold change ≥ 1.5. The top DEGs related to nitrogen metabolism were selected for this analysis.

### Validation of DEGs Using Quantitative Real-Time PCR

To validate the RNA-seq data, quantitative reverse transcription PCR (qRT-PCR) analyses of selected genes were performed. The total RNA was extracted from S. *dextrinosolvens* Z6 grown in different nitrogen sources and reverse transcribed into cDNA by using first-strand cDNA Synthesis Kit (Genenode Biotech, Beijing). Quantitative PCR was performed using SYBR green (Genenode Biotech, Beijing, China) by using a FS384 real-time PCR system (Changzhou Fusheng Biotechnology, China) in a 10 μl mixture containing DNA template 2 μl, forward and reverse primer 0.25 μl each, 2 × SYBR Green qPCR Mix (Antibody) 5 μl, and DEPC-ddH_2_O 2.5 μl. The PCR amplification began with a 2 min denaturing step at 94°C, followed by 40 cycles at 94°C for 15 s, and 60°C for 30 s of annealing extension. The primers designed for RT-qPCR in this study are listed in Supplementary Table 10. The relative quantification of a gene is defined as the change in expression of the target gene relative to the reference genes. The relative expression level of each gene was calculated by the formula (2^–ΔΔ*CT*^).

### Statistical Analysis

The enzyme activities (urease, GDH, GS, and GOGAT) and MCP data were analyzed using the mixed model, and real-time PCR data were analyzed by one-way ANOVA in SAS 9.2 (SAS Institute SAS/STAT(R), 2009). The model included fixed effects of time and nitrogen sources (ammonia, amino acid, and urea), and the results are presented as histograms made using GraphPad Prism version 8.0.0 for Windows, GraphPad Software, San Diego, California, United States^3^. Least square means are reported, and the significance was set to *P* < 0.05. The correlations between the activity of the analyzed enzymes and the expression of the corresponding DEGs were described by using Pearson’s correlation coefficient.

### Data Availability

The complete genome sequence for *S. dextrinosolvens* strain Z6 has been deposited in GenBank under the accession number CP047056, BioProject number PRJNA545926, and BioSample number SAMN13552832. The raw genome sequence data are available in the Sequence Read Archive (SRA) database under accession number SRR10680263. The raw data for transcriptome sequencing are available in the same Bioproject number, Biosample number SAMN15009434, and in the SRA database under accession number SRR11855358-SRR11855371.

## Results

### Complete *S. dextrinosolvens* Z6 Genome Sequence

Following enrichment and spreading on plates, a single bacterial colony was isolated based on its morphological growth (red color) on the solid M3 media. PCR amplification using urease gene primers with DNA from the selected colony as a template resulted in a clear band, and extracts derived from cells from the colony had urease activity (85.4 ± 1.77 NH_3_ nmol/min/mg protein). Furthermore, 16S rRNA sequencing with subsequent comparison to NCBI database sequences using BLAST revealed that the selected colony showed the highest identity to *S. dextrinosolvens* strain 0554 (98.13% identity). We named the strain isolated here, *S. dextrinosolvens* Z6.

**FIGURE 1:**
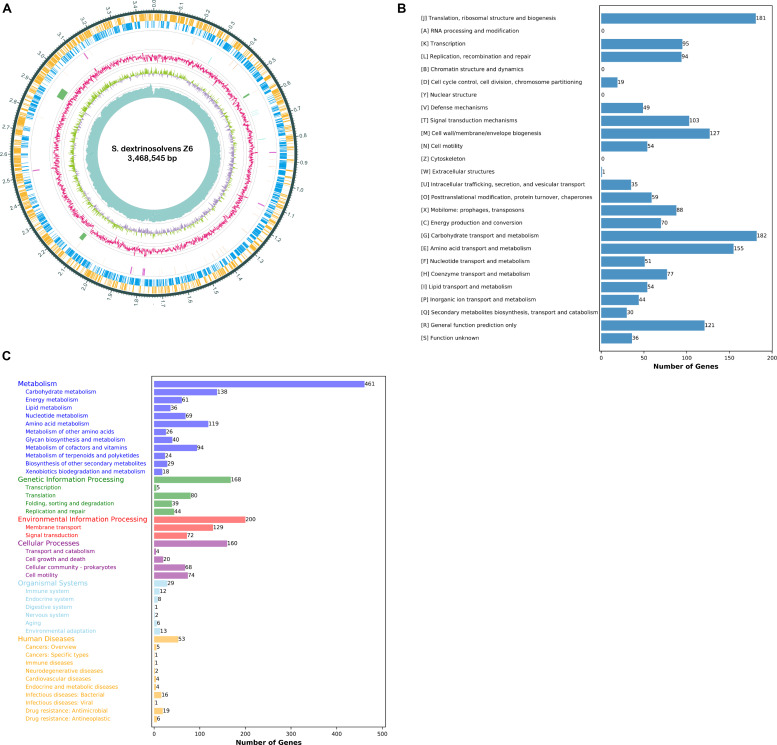
*Succinivibrio dextrinosolvens* strain Z6 genome information. **(A)** Circle diagram showing the nuclear genome. From the outside to the inside are the coding genes (sense), coding genes (antisense), tRNAs (orange) and rRNAs (purple), CRISPRs (blue) and gene islands (green), GC ratio, GC-skew, and sequencing depth. **(B)** Functional classification of genomically encoded proteins in clusters of orthologous groups (COG). **(C)** Functional classification of genomically encoded proteins using the Kyoto Encyclopedia of Genes and Genomes (KEGG) database.

We determined that the *S. dextrinosolvens* Z6 genome is 3.47 Mbp, with a G + C content of 38.9% (Figure 1A), a total of 2993 coding sequences, 21 rRNA genes (7 copies of each 5S rRNA, 16S rRNA, and 23S rRNA), 69 tRNAs, 7 ncRNAs, 3 gene islands, and 5 CRISPR repeats (Table 1). Functional annotation of the genome indicated that the percentage of protein-encoding genes was 98%, while 1268 genes (42.4% of the total protein-encoding gene) are of unknown function. The genes with an annotated function included those involved in “regulating carbohydrate transport and metabolism” (182 genes), “translation, ribosomal structure and biogenesis” (181), accounting for ∼10% each, and “regulation of amino acid transport and metabolism” (155). KEGG annotation indicated that most of the annotated genes were involved in carbohydrate metabolism (138 genes), membrane transport (129 genes), and amino acid metabolism (119 genes) (Figures 1B,C).

**TABLE 1:** Statistics for genomic structure prediction of *S. dextrinosolvens* Z6.

Type	Number	Length (bp)	% genome
tRNA	69	5434	0.16
16S rRNA	7	10,446	0.30
23S rRNA	7	20,039	0.58
5S rRNA	7	805	0.02
CDS	2,993	2,952,486	85.12
CRISPR	5	4382	0.13
Genomic_island	3	77,869	2.25

### Comparative Genome Analysis of *S. dextrinosolvens* Strains

**FIGURE 2:**
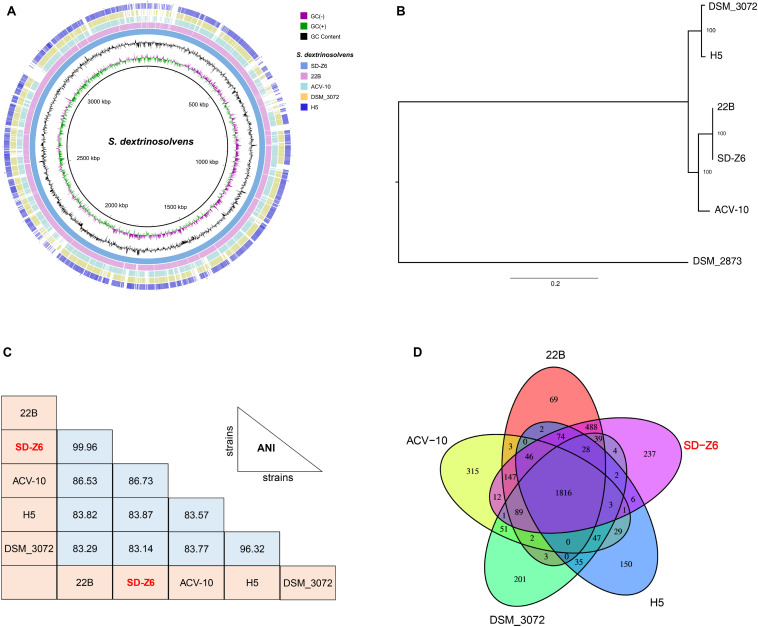
Comparative genome analysis of *Succinivibrio dextrinosolvens* strain Z6 and closely related strains. **(A)** BLAST Ring Image Generator (BRIG) analysis of *S. dextrinosolvens* strain Z6 and the genomes of *S. dextrinosolvens* 22B, *S. dextrinosolvens* ACV-10, *S. dextrinosolvens* H5, and *S. dextrinosolvens* DSM_3072. The innermost ring represents the skew (- and +), followed by GC content. The third ring shows *S. dextrinosolvens* strain Z6 (blue), ring 4 *S. dextrinosolvens* 22B (pink), ring 5 *S. dextrinosolvens* ACV-10 (light blue), ring 6 *S. dextrinosolvens* DSM_3072 (yellow), and ring 7 *S. dextrinosolvens* H5 (dark blue). **(B)** Phylogenetic tree highlighting the relationship of *S. dextrinosolvens* strain Z6 with other strains in the *Succinivibrio* genus. The evolutionary history was inferred using the maximum likelihood method and showed that *S. dextrinosolvens* Z6 is a member of the *Succinivibrio* genus. The percentage of trees in which the associated taxa clustered together is shown next to the branches. *Succinimonas amylolytica* DSM_2873 is an outgroup. **(C)** Pairwise comparisons of average nucleotide identity (ANI), and **(D)** global gene conservation in *Succinivibrio*. Each oval represents the total number of genes in each genome. Overlapping regions depict the number of genes shared between the respective genomes.

Circular genome comparison (Figure 2A) of *S. dextrinosolvens* Z6 with related strains in the genus revealed examples of dissimilarity, such as gaps, indicating the uniqueness of this strain, and a whole genome alignment between *S. dextrinosolvens* Z6 and the very closely related *S. dextrinosolvens* 22B showed more than 0.34 Mb of nucleotide polymorphisms (gaps) (Supplementary Table 1). The strain isolated here was fully sequenced, whereas the other strains in the genus are only represented by partial genome sequences (Supplementary Table 1). A phylogenetic tree analysis of *S. dextrinosolvens* strains showed that strain Z6 was most closely related to *S. dextrinosolvens* 22B (Figure 2B). ANI values between *S. dextrinosolvens* strain Z6 and *S. dextrinosolvens* 22B, *S. dextrinosolvens* ACV-10, *S. dextrinosolvens* H5, and *S. dextrinosolvens* DSM_3072 were 99.96% (64.09% genome coverage), 86.73% (44.24% genome coverage), 83.87% (39.89% genome coverage), and 83.14% (41.14% genome coverage), respectively (Figure 2C and Supplementary Table 2), which is above the recommended cutoff for species delineation (<95–96% ANI) for *S. dextrinosolvens* 22B.

*S. dextrinosolvens* Z6 had the highest gene and protein number of strains reported from the genus (Supplementary Table 1). A comparative gene-by-gene distribution analysis (Figure 2D) between *S. dextrinosolvens* Z6, *S. dextrinosolvens* 22B, *S. dextrinosolvens* ACV-10, *S. dextrinosolvens* H5, and *S. dextrinosolvens* DSM_3072 showed that 1816 genes (61%) were conserved among each *Succinivibrio* genomes. Furthermore, 237 genes were unique to *S. dextrinosolvens* Z6, compared to 69 for *S. dextrinosolvens* 22B, 315 for *S. dextrinosolvens* ACV-10, 150 for *S. dextrinosolvens* H5, and 201 for *S. dextrinosolvens* DSM_3072. Thus, the genome sequence of *S. dextrinosolvens* Z6 adds considerable information about the genetic diversity within the *Succinivibrio* genus.

### *S. dextrinosolvens* Z6 Genes Involved in Nitrogen Metabolism

**FIGURE 3:**
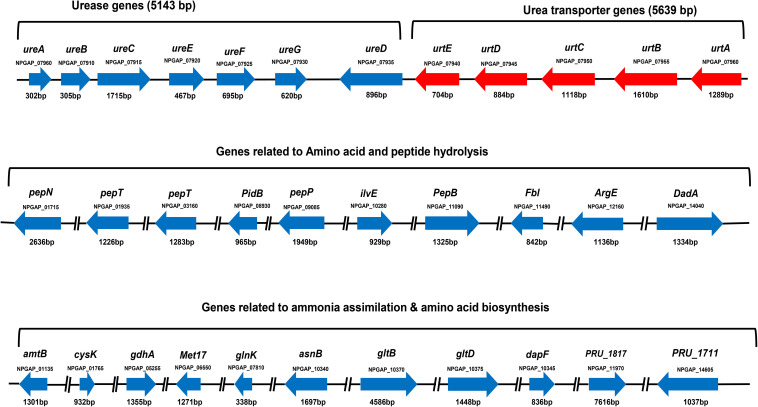
Gene clusters for urea-nitrogen hydrolysis and transportation; gene groups for amino acid and peptide degradation; and ammonia assimilation and amino acid biosynthesis. The code written under the gene represents locus tag. The solid line between the genes indicates the genes on the same cluster and the broken line indicates the genes found in different clusters.

Nitrogen metabolism–related genes in the genome of strain Z6 were grouped as genes involved in urea hydrolysis, amino acid- and peptide-degrading genes, and genes involved in ammonia assimilation and amino acid biosynthesis (Figure 3). The open read frames (ORFs) for the urea hydrolysis-related urease (*ureA*, *B*, *C*, *E*, *F* and *D*) and the urea transporter (*urtE*, *D*, *C*, *B*, and *A*) genes, which encode a protein of urease enzyme and urea transporter, respectively, clustered together and involved in urea hydrolysis (Table 2). The genes involved in amino acid and peptide hydrolysis, such as *pepN*, *T*, *P* and *B*, *PldB*, *ilvE*, *fbl*, *ArgE*, and *DadA*, were found to be scattered throughout the genome. They encode enzymes that degrade proteins, peptides, and amino acids at different structural sites. Genes involved in ammonia assimilation and the biosynthesis of amino acids (*asnB*, *gltBD*, and *dapF*), such as alanine, aspartate, glutamate, and lysine, were also identified in the genome. However, other genes known to be involved in regulation of nitrogen metabolism and amino acid biosynthesis were spread throughout the genome.

**TABLE 2:** The comparison between *Succinivibrio dextrinosolvens* strains in urease subunit and urea transporter genes.

Strains of *S. dextrinosolvens*	Urease genes	Urea transporter genes
Z6	+	+
22B	+	+
ACV-10	−	+
H5	−	−
DSM_3072	−	−

**+ represent present and − denote absent.**

### Phenotypic and Chemotaxonomic Characterization

**FIGURE 4:**
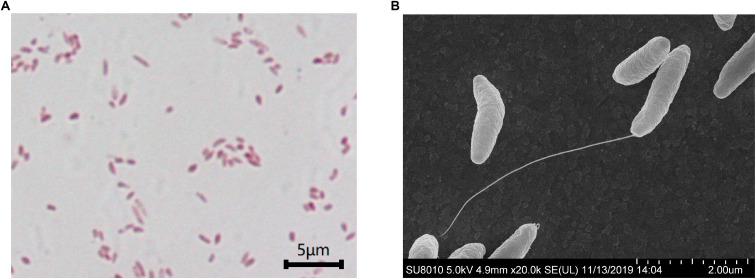
Scanning electron microscopy images of *Succinivibrio dextrinosolvens* strain Z6. **(A)** Gram staining of *S. dextrinosolvens* strain Z6. **(B)** Scanning electron micrographs of *S. dextrinosolvens* strain Z6. Scale as indicated on the respective panel.

**FIGURE 5:**
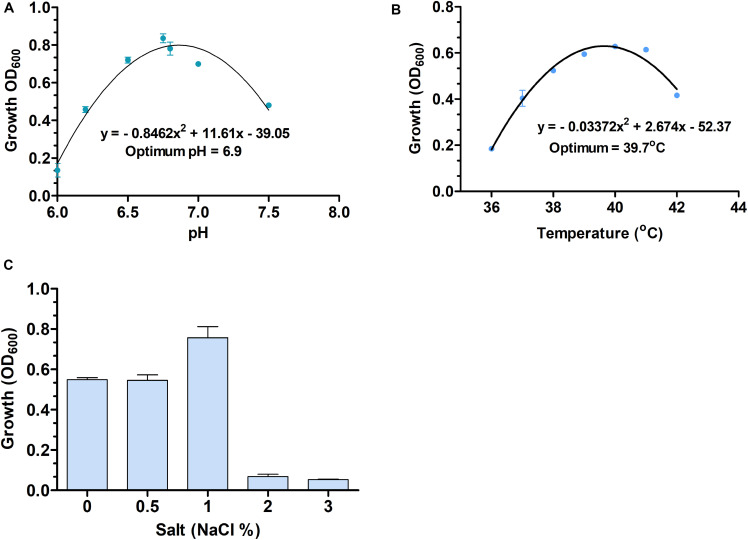
The optimum temperature and pH **(A,B)**, and different salt (% NaCl) concentrations **(C)** used for *Succinivibrio dextrinosolvens* strain Z6 growth. Data points represent averages from triplicate experiments. Error bars represent standard deviations.

*S. dextrinosolvens* Z6 is a strictly anaerobic, gram-negative bacterium with rod-shaped cells 0.12–0.47 μm × 1.01–1.7 μm (width × length). The cells are found singly or in pairs as shown by scanning electron microscopy (SEM) (Figures 4A,B) and have the basic morphological characteristics reported for other members of the *Succinivibrio* genus. Growth takes place at temperatures from 36 to 42°C with an optimum of 39.7°C (Figure 5A), and a pH range from 6 to 8, with an optimum of 6.9 (Figure 5B). Supplementation with 0–1% (w/v) NaCl supported growth, with maximum growth at 1% (Figure 5C).

The predominant detected polar lipids in *S. dextrinosolvens* Z6 were phosphatidyl glycerol (PG), phosphatidyl ethanolamine (PE), unidentified aminophospholipid (UAPL), and unidentified lipids (UL 1–4), and the cellular fatty acid profile showed that C16:0 FAME (35.34%) and summed feature 10 (C18:1 c11/t9/t6 FAME) (30.93%) were the major components. The major cell wall sugars were ribose and glucose, and the detected DAP (2, 6-diaminopimelic acid) composition in the cell wall was meso-DAP.

A carbohydrate utilization assay revealed that *S. dextrinosolvens* Z6 fermented *D*-glucose, *D*-xylose, maltose, *L*-arabinose, esculin, *D*-sorbitol, *D*-cellobiose, and *L*-rhamnose, while trehalose, lactose, starch, cellulose, glycerol, inositol, and *D*-melezitose were not fermented (Supplementary Table 2). API ZYM strips showed that *S. dextrinosolvens* Z6 was positive for the alkaline phosphatase, esterase lipase, α-chymotrypsin, acid phosphatase, naphthol-AS-BI-phosphohydrolase, leucine arylamidase, valine arylamidase, cysteine arylamidase, and N-acetyl-β-glucosaminidase enzymes, while lipase, trypsin, galactosidase (α and β), glucosidase (α and β), β-glucuronidase, and α-fucosidase tests were negative. A MALDI-TOF spectrum of our strains was compared with spectra in the database and the score values of our strain was less than 1.7 as shown in Supplementary Figure 1.

### Nitrogen Metabolism of *S. dextrinosolvens* Z6 Grown With Different Nitrogen Sources

**FIGURE 6:**
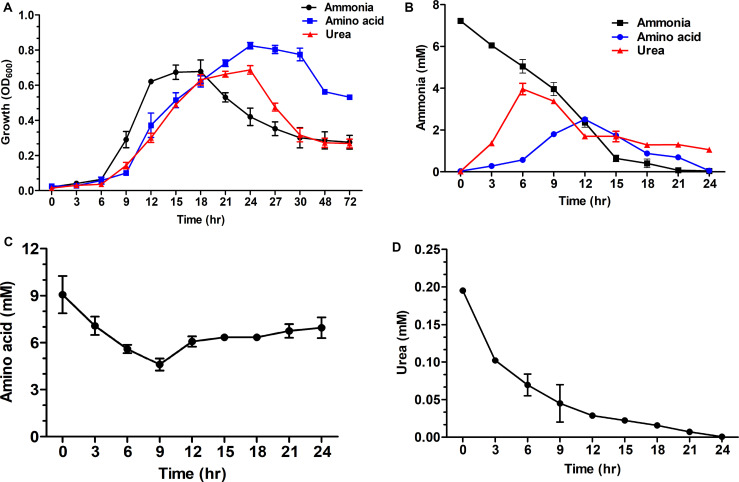
Time course measurements of the effect of nitrogen sources on growth of *S. dextrinosolvens* Z6. Data points represent averages for triplicate cultures. Error bars represent standard deviations. **(A)** Growth. **(B)** Ammonia utilization and production. **(C)** Amino acid utilization. **(D)** Urea utilization.

*S. dextrinosolvens* Z6 was grown in media containing either urea, ammonia, or amino acids as a nitrogen source, and preferential amino acid utilization was observed (Figure 6A). The maximum growth rate on different nitrogen sources during the exponential growth phase was lower with urea (0.065 ± 0.006 h^–1^) and amino acids (0.091 ± 0.026 h^–1^) than with ammonia (0.11 ± 0.016 h^–1^). The maximum cell density at OD_600_ was 0.825 ± 0.018, 0.697 ± 0.056, and 0.687 ± 0.024 for growth on amino acid, ammonia, and urea, respectively (Supplementary Table 4). The concentrations of ammonia and urea used by *S. dextrinosolvens* Z6 were inversely related to the growth phase of the bacteria, whereas the level of amino acid utilization showed a small decline, and after 9 h of incubation, we observed a slight increase and a subsequent stationary profile (Figures 6B–D). The concentration of ammonia in the medium supplemented with ammonia sulfate showed a continuous decrease throughout the exponential growth phase and was almost depleted within 21 h. During growth in urea, concentrations of ammonia increased between 3 and 6 h (Figure 6B), with a continuous decrease in urea concentration (Figure 6D). Finally, the ammonia concentration in the medium supplemented with amino acids was shown to increase between 6 and 12 h, but slower than the rate of ammonia produced by urea.

### *S. dextrinosolvens* Z6 Ammonia Assimilation in Different Nitrogen Sources

**FIGURE 7:**
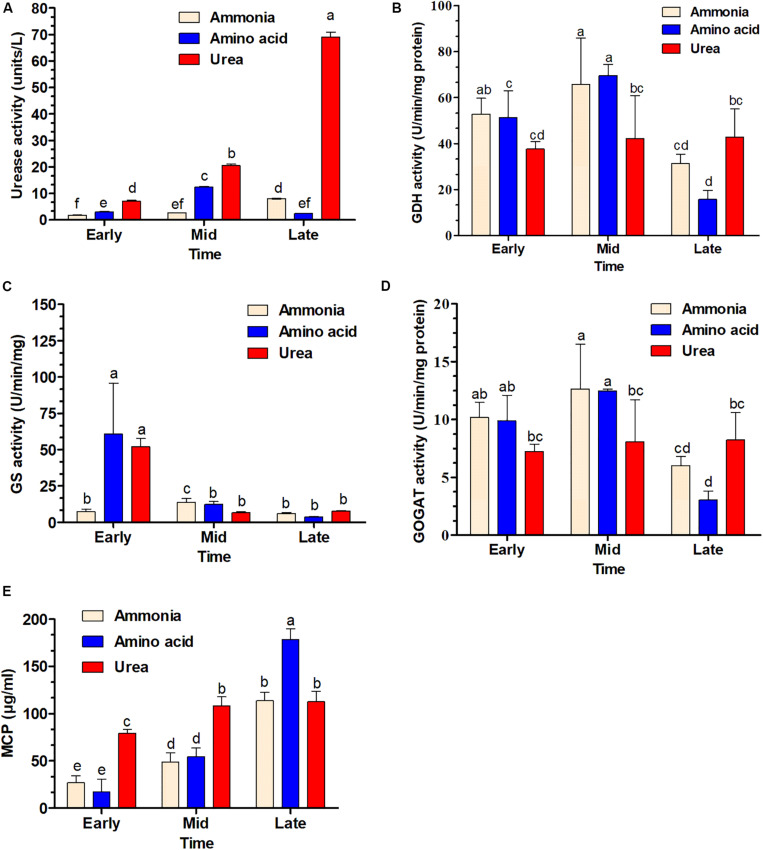
Time course determination of enzyme activity and soluble microbial crude protein production (MCP) in *S. dextrinosolvens* Z6 grown on different nitrogen sources. The bars represent averages for triplicate cultures used to measure enzyme activity. Error bars represent standard deviations. **(A)** Urease activity. **(B)** Glutamate dehydrogenase (GDH) activity. **(C)** Glutamine synthetase (GS) activity. **(D)** Glutamate synthase (GOGAT) activity. **(E)** Soluble microbial crude protein. The data points represent early, mid, and late exponential growth phases. Bars with different letters are significantly different.

Urease (*P* < 0.01), GS (*P* < 0.01), and GDH (*P* < 0.05) activities were significantly different in response to different nitrogen sources, while GOGAT activity was not significantly different (*P* = 0.26) for different nitrogen sources. However, all enzyme activities were significantly different (*P* < 0.01) in the three growth phases. The results for the early and mid-exponential growth phases showed that urease and GS activities were low when cells were grown in a medium with ammonia. These enzyme activities were far higher for the bacteria grown in medium supplemented with urea and amino acids, especially during the earlier growth phase for GS (Figure 7C). In contrast, GDH activity was significantly higher for cells grown in ammonia-containing medium. Notably, urease activity was higher for bacteria grown in urea-containing medium in the late exponential growth phase and a continuous increase was shown throughout the growth phase. The cultures with ammonia and amino acids were shown to have the highest GDH and GOGAT activities in the mid-exponential growth phase (Figures 7B,D). The MCP was significantly different (*P* < 0.01) for both different nitrogen sources and growth phases.

### RNA Sequencing and Read Mapping

Fifteen libraries were established with the following designations: AA1, AA2, AA3, AA4, and AA5 were from amino acid supplement; AMO1, AMO2, AMO3, AMO4, and AMO5 were ammonia supplement, while U1, U2, U3, U4, and U5 were urea-supplemented groups with five biological replicates, respectively. The cDNA libraries were prepared and sequenced by Illumina HiSeq platform, which generated a total of 812,540,682 raw reads (Supplementary Table 5). After removing the adaptors and low-quality reads, 787,498,536 clean reads were obtained with a high quality of Q30_96.23%. We then mapped the trimmed clean reads onto the reference genome and 36.98–85.64% of the clean reads were mapped uniquely to the genome, whereas CDs mapped reads 26.88–86.18% onto the genome (Supplementary Table 6).

### Transcriptome Analysis in Response to Growth on Different Nitrogen Sources

**FIGURE 8:**
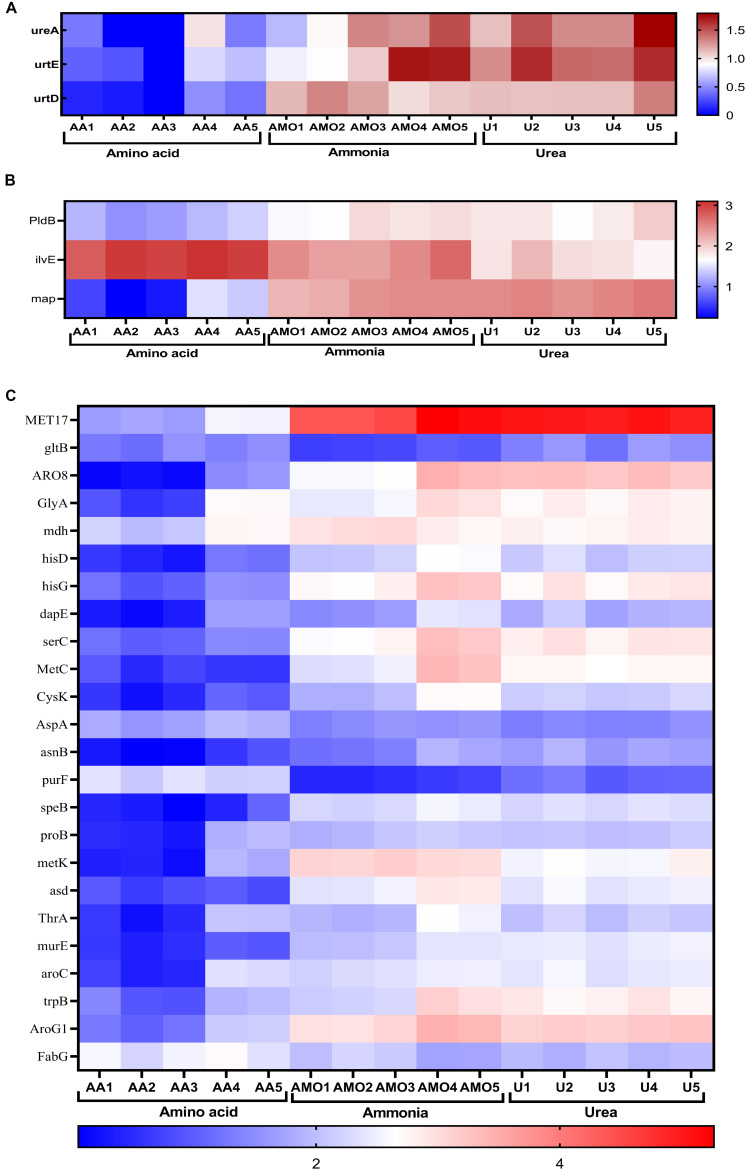
Comparison of differentially expressed genes in cells grown in media containing amino acids, ammonia, or urea. Heat map displaying log-transformed normalized transcript abundances for a subset of genes involved in nitrogen metabolism. **(A)** Urease and urea transporter genes. **(B)** Genes involved in amino acid and peptide degradation. **(C)** Genes involved in ammonia assimilation and amino acid biosynthesis. Abbreviations AA1-5, AMO1-5, and U1-5 represent the five replications of amino acid, ammonia, and urea, respectively.

*S. dextrinosolvens* Z6 showed differential gene expression patterns during growth in different nitrogen sources. A total of 1246 genes displayed statistically significant fold changes in their transcript abundances during growth on amino acids, ammonia, or urea. Among the DEGs were 33 related to nitrogen metabolism (Figures 8A–C). Genes (*ilvE*, *CysK*, *PurF*, and *FabG*) related to amino acid degradation and biosynthesis (mainly branched chain amino acid aminotransferase; enzymes involved in cys/met metabolism, and in phenylalanine, tyrosine, and tryptophan biosynthesis) were significantly differentially expressed in cells grown on amino acids than on urea and ammonia. The highest detected log fold changes in transcript abundances corresponded to the clustered genes encoding the branched chain amino acid aminotransferase (2.55 log-fold changes) followed by 3-deoxy-7-phosphoheptulonate synthase and pyridoxal-phosphate-dependent enzymes involved in amino acid biosynthesis (Supplementary Table 7).

### Quantitative Real-Time PCR Validation

In this study, a total of eight nitrogen metabolism-related candidate genes found to be differentially expressed in *S. dextrinosolvens* Z6 grown in a medium containing urea, ammonia, or amino acid were selected for validation to understand their expression level. Quantitative real-time PCR analysis reveals that all the eight assessed candidate genes were differentially expressed in relation to the type of nitrogen sources (Supplementary Figure 2).

## Discussion

Nitrogen is an essential macronutrient for microbial growth and basic metabolic processes and nitrogen sources utilized by different ruminal bacteria include urea, free amino acids, and peptides as non-protein nitrogen-containing compounds (NPN), and true proteins. The degradation and utilization rate of nitrogen sources can vary depending on the species and strain of bacteria. Accordingly, identifying the representative bacteria and understanding their role in the utilization of different nutrients available will provide knowledge that can help manipulate different related microbes for better utilization of feed resources.

In the present study, the ureolytic bacterium *S. dextrinosolvens* Z6 was isolated from rumen fluid samples collected from dairy cows fed with roughage and concentrate mix. To enrich and analyze its urease-producing capacity, a urea-containing medium was used since, as previously described (Jin et al., 2016); a color change in the medium supplemented with indicators can identify urease-producing organisms (Moore, 1977). Other isolates of this species have previously been obtained from rumen of sheep, cattle, and wallaby guts (Bryant and Small, 1955; Morrison et al., 2011), and have shown significant differences in terms of nitrogen utilization and physiological requirements. The isolated strain was examined morphologically by SEM and was found to be much shorter than the strain isolated by Bryant and Small (1955). Strain 22B and our strain Z6 have clusters of urease and urea transporter genes (Figure 7), which is an indication for the potential of this bacteria in utilizing urea as a nitrogen source as shown by Bryant and Small (1955).

The effect of different nitrogen sources on *S. dextrinosolvens* Z6 growth was examined by comparing *in vitro* growth on ammonia, amino acid, and urea as sole nitrogen sources (Figure 6A). *S. dextrinosolvens* Z6 preferentially utilized amino acids, which is consistent with a previous report (Gomez-Alarcon et al., 1982). Although the maximum growth was lower than for amino acids, strain Z6 was also able to grow on ammonia and urea, and showed a relatively faster growth rate on ammonia. This distinguishes strain Z6 from other reported strains, which prefer ammonia as the nitrogen sources for optimal growth. This was confirmed by the genome sequence and transcriptome analysis, in that strain Z6 contained genes encoding enzymes such as urease, deaminase, aminotransferase and peptidase, which are involved in urea, amino acid, and peptide utilization and metabolism.

In this study to keep RNA integrity/quality, bacteria growth and further enzymatic action have stopped immediately at the time of mid-exponential growth phase; we have used 4°C for harvesting bacteria pellet by centrifuge, but this temperature might cause cold shock response by the bacteria that affect genes’ transcriptional expression (Gao et al., 2006; Liu et al., 2014; Rastrojo et al., 2019). However, we have limited the time to 5 min as it has been applied by Liu et al. (2014) which may reduce this effect on the RNA expression. The enzyme branched-chain amino acid aminotransferase encoded by *ilvE* catalyzes the conversion of branched-chain amino acids (L-leucine, L-isoleucine, and L-valine) and α-ketoglutarate into branched chain α-keto acids and glutamate. This enzyme was upregulated in cells grown in amino acid-containing media. This suggests that *S. dextrinosolvens* Z6 can utilize branched chain amino acids for growth and is consistent with studies showing that ruminal bacteria are inhibited and yields of microbial protein decreased in goats fed on a diet from which all branched chain amino acids (BCAA) were removed (Wang et al., 2008; Apajalahti et al., 2019). Furthermore, as has been indicated by different studies, this enzyme is vital for the growth and survival of bacteria (Zhang et al., 2013; Amorim Franco and Blanchard, 2017; Li et al., 2017). However, the level of free amino acids declined for the first 9 h of incubation and subsequently was constant, which might be explained by the production of different nitrogen-containing compounds in the medium. Consistent with this idea, other studies have shown that bacteria can produce some amino acids and release them to the surrounding environment (Lam et al., 2009; Aliashkevich et al., 2018).

We observed an increased transcript abundance of the glutamine phosphoribosylpyrophosphate amidotransferase encoding gene (*purF*) during growth in amino acid-containing media, unlike with the other two media, which suggests that *S. dextrinosolvens* Z6 can use *L*-glutamine to produce purine via the purine biosynthesis pathway (Yee et al., 2015). Purine is a precursor for GTP synthesis and thus plays an important role in energy production and growth regulation (Kriel et al., 2012).

Urease activity was higher in all growth phases in the presence of urea than in the presence of ammonia or amino acids. This is also consistent with the transcript abundance of the urease gene structural components (*ureA*) and the urea transporter protein-encoding genes, *urtE* and *urtD*, which were expressed at higher levels in the presence of urea. A combination of the two gene clusters would support bacterial growth in the urea-containing medium and expand the range of potential nitrogen sources. This result revealed that strain Z6 utilizes an energy-dependent system including the urea transporter, which is encoded by the *urtABCDE* gene cluster, for taking up urea for hydrolysis. This is consistent with certain other bacterial isolates (Beckers et al., 2004) in which the transporter genes are transcribed in response to nitrogen limitation.

When *S. dextrinosolvens* Z6 was grown in ammonia, we observed higher glutamate dehydrogenase (NADH-GDH) activity compared to growth on amino acids and urea. However, the transcript of the gene related to GDH was not detectable during growth on any nitrogen source. The activity of GS during the early exponential growth phase was significantly higher for cells grown on amino acids and urea, likely due to limited ammonia levels, since previous studies reported that the activity and expression of GS and its gene were higher when cells were grown at low ammonia concentrations (Hua et al., 2004; Kim et al., 2012).

*HisD* and *hisG* are involved in the biosynthesis of histidine in bacteria and other organisms and were expressed at higher levels as a result of growth in urea as compared to growth in ammonia-containing media. These genes encode the protein product of histidinol dehydrogenase and ATP phosphoribosyltransferase, respectively, and were among the DEGs that were more highly expressed. However, other studies have reported that the presence of urea inhibits the activities of these enzymes (Yourno, 1968; Andorn and Aronovitch, 1982; Senior, 1989; Mizukami et al., 1994).

It is evident from the results of this current study that different nitrogen sources affect the enzyme activity and transcript abundance of certain proteins and genes in *S. dextrinosolvens* Z6, suggesting that there is potential for regulating its nitrogen metabolism. Because of the availability of different nitrogen sources, concentration, and passage rate, it is hard to make a direct comparison between *in vitro* experiment and real ruminal activities. However, this finding gives a basis to understand the role of the whole ruminal bacteria community.

## Data Availability Statement

All datasets presented in this study are included in the article/Supplementary Material.

## Ethics Statement

The animal study was reviewed and approved by the Ethics Committee of Institute of Animal Sciences of CAAS (No. IAS2019-14).

## Author Contributions

SH and SZ equally contributed to this study’s conceptualization, methodology, software handling, formal analysis, investigation, writing – original draft preparation, and writing – review and editing. JW contributed to validation, supervision, project administration, and funding acquisition. All authors have read and agreed to the published version of the manuscript.

## Conflict of Interest

The authors declare that the research was conducted in the absence of any commercial or financial relationships that could be construed as a potential conflict of interest.

## Footnotes

1 http://www.bioinformatics.babraham.ac.uk/projects/fastqc/

2 https://github.com/jstjohn/SeqPrep

3 www.graphpad.com
