# Supplementary material for: Complete Genome Sequencing and Transcriptome Analysis of Nitrogen Metabolism of Succinivibrio dextrinosolvens Strain Z6 Isolated From Dairy Cow Rumen
Source: Front Microbiol. 2020 Aug 14;11:1826. doi: 10.3389/fmicb.2020.01826 (PMC7507024; doi:10.3389/fmicb.2020.01826)
Supplement: Supplementary file 1 [file Data_Sheet_1.DOCX]

Supplementary Material

## Supplementary Figures


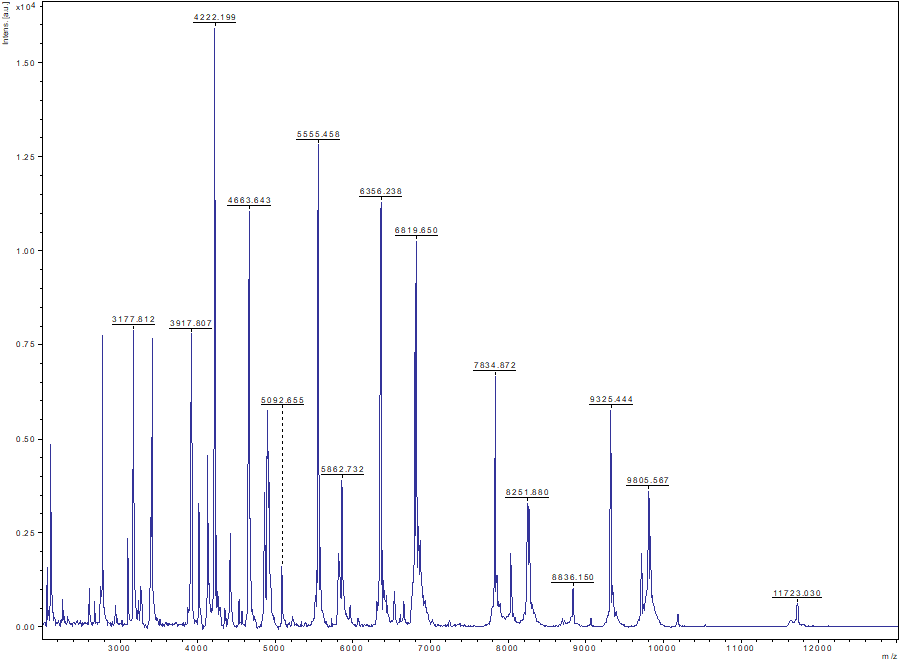


**Supplementary Figure 1.** MALDI-TOF MS analysis of *S. dextrinosolvens* Z6. Reference mass spectrum from the *S. dextrinosolvens* Z6

**Supplementary Figure 2.** Relative expression of genes validated by qRT-PCR

**Supplementary Tables**

**Supplementary Table 1**. Comparative genomic characteristics of the five members of genus *Succinivibrio*

| **Organism/Name** | **Strain** | **Size (Mb)** | **GC%** | **Scaffolds** | **Genes** | **Proteins** | **References** |
| --- | --- | --- | --- | --- | --- | --- | --- |
| *Succinivibrio dextrinosolvens* | Z6 | 3.46855 | 38.9 | 1 | 3091 | 2993 | This study |
| *Succinivibrio dextrinosolvens* | DSM 3072 | 2.66678 | 39.0 | 104 | 2397 | 2247 | Varghese, 2017 |
| *Succinivibrio dextrinosolvens* | H5 | 2.67547 | 39.0 | 106 | 2392 | 2244 | Varghese, 2016 |
| *Succinivibrio dextrinosolvens* | ACV-10 | 2.96174 | 39.7 | 94 | 2624 | 2484 | Varghese, 2016 |
| *Succinivibrio dextrinosolvens* | 22B | 3.12695 | 38.8 | 178 | 2819 | 2604 | Kelly *et al*., 2014 |

**Supplementary Table 2.** Genome coverage of ANI Results for *S. dextrinosolvens* strains

| **Strains comparison (A vs B)** | **Metric** | |
| --- | --- | --- |
|  | **Genome A coverage (%)** | **Genome B coverage (%)** |
| Z6 vs. 22B | 64.09 | 72.97 |
| Z6 vs. ACV-10 | 44.24 | 52.61 |
| Z6 vs. DSM_3072 | 41.14 | 54.59 |
| Z6 vs. H5 | 39.89 | 52.84 |
| H5 vs. 22B | 51.34 | 44.14 |
| H5 vs. ACV-10 | 53.42 | 47.97 |
| H5 vs. DSM_3072 | 60.71 | 60.83 |
| DSM_3072 vs. 22B | 50.41 | 43.25 |
| DSM_3072 vs. ACV-10 | 54.13 | 48.51 |
| ACV-10 vs. 22B | 53.83 | 51.54 |

**Supplementary Table 3.** Biochemical results of *S. dextrinosolvens* Z6

| **Carbohydrate** |  | **Results** |
| --- | --- | --- |
| D-glucose |  | **+** |
| D-mannitol |  | **+** |
| D-lactose |  | **-** |
| D-saccharose (sucrose) |  | **+** |
| D-maltose |  | **+** |
| Salicin |  | **+** |
| D-xylose |  | **W** |
| L-arabinose |  | **+** |
| Gelatin (bovine origin) |  | **-** |
| Glycerol |  | **-** |
| D-Cellobiose |  | **+** |
| D-mannose |  | **-** |
| D-melezitose |  | **-** |
| D-raffinose |  | **-** |
| Starch |  | **-** |
| Cellulose |  | **-** |
| D-sorbitol |  | **+** |
| L-rhamnose |  | **+** |
| D-trehalose |  | **-** |
| **API ZYM** | | |
| Alikaline phosphatase |  | **-** |
| Esterase (C4) |  | **+** |
| Esterase Lipase(C8) |  | **+** |
| Lipase (C14) |  | **+** |
| Leucine arylamidase |  | **+** |
| Valine arylamidase |  | **+** |
| Cystine arylamidase |  | **-** |
| Trypsin |  | **-** |
| α-chymotrypsin |  | **-** |
| Acid phosphatase |  | **+** |
| Naphthol-AS-BI-phosphohydrolase |  | **+** |
| α-galactosidase |  | **-** |
| β-galactosidase |  | **-** |
| β-glucuronidase |  | **-** |
| α-glucosidase |  | **-** |
| β-glucosidase |  | **-** |
| N-acetyle-β-glucosaminidase |  | **+** |
| α-mannosidase |  | **-** |
| α-fucosidase |  | **-** |

Note: - **^+^** represent the bacteria can ferment, **^-^** not ferment and **^w^** weakly ferment for carbohydrate test; **^+^** present and **^–^** absent for enzyme activities

**Supplementary Table 4.** Effects of different nitrogen sources on growth of *S. dextrinosolvens* Z6

| **Nitrogen sources** | **Maximum growth density (OD_600_)*** | **Growth rate (h^-1^)**** |
| --- | --- | --- |
| Ammonia | 0.697±0.056 | 0.11±0.016 |
| Amino acid | 0.825±0.018 | 0.091±0.026 |
| Urea | 0.687±0.024 | 0.065±0.006 |

*The maximum optical density over 72hrs of incubation; ** the maximum specific growth rate during exponential growth phase. The value is mean ± standard deviation of five technical replicates.

**Supplementary Table 5**. Summary of sequence assembly after Illumina sequencing

| **Sample Name** | **Raw reads** | **Clean Reads** | **Clean Error Rate (%)** | **Clean Q30 (%)** | **rRNA Ratio(%)** | **none rRNA(bp)** |
| --- | --- | --- | --- | --- | --- | --- |
| AA1 | 49566992 | 48380288 | 0.0114 | 96.13 | 14.029 | 878,611,354 |
| AA2 | 42036780 | 41165326 | 0.0121 | 95.31 | 41.057 | 2,228,263,583 |
| AA3 | 40121296 | 39167196 | 0.0114 | 96.23 | 16.717 | 834,878,323 |
| AA4 | 42947006 | 41900662 | 0.012 | 95.41 | 16.638 | 936,217,909 |
| AA5 | 39768212 | 38823886 | 0.0122 | 95.25 | 19.927 | 1,037,279,792 |
| AM01 | 51449570 | 50534088 | 0.0123 | 95.08 | 11.094 | 757,683,230 |
| AM02 | 78819786 | 73940456 | 0.0129 | 94.4 | 14.005 | 1,316,869,441 |
| AM03 | 66749076 | 65535902 | 0.0115 | 96.06 | 14.735 | 1,223,861,999 |
| AM04 | 68421528 | 64112638 | 0.0128 | 94.52 | 7.328 | 641,426,002 |
| AM05 | 66854988 | 62744904 | 0.0129 | 94.35 | 7.168 | 616,794,012 |
| U1 | 49114086 | 48191230 | 0.0119 | 95.58 | 14.054 | 905,769,756 |
| U2 | 55489622 | 54546292 | 0.0118 | 95.67 | 11.447 | 848,272,022 |
| U3 | 50672058 | 49627764 | 0.012 | 95.45 | 10.813 | 714,782,444 |
| U4 | 53121520 | 52186088 | 0.012 | 95.46 | 9.812 | 695,215,638 |
| U5 | 57408162 | 56641816 | 0.0116 | 95.92 | 10.339 | 788,649,224 |

AA1-AA5: amino acid; AMO1-AMO5: ammonia and U1-U5: urea as nitrogen sources. Q30: The percentage of bases with a Phred value > 30

**Supplementary Table 6**. Summary of clean reads mapped to the reference genome

| **Sample Name** | **Total reads** | **Uniq Mapped Reads** | **CDS Mapped Reads** |
| --- | --- | --- | --- |
| AA1 | 48380288 (97.84%) | 31471279 (65.05%) | 19394342 (40.09%) |
| AA2 | 41165326 (61.48%) | 15224740 (36.98%) | 11066440 (26.88%) |
| AA3 | 39167196 (97.61%) | 22685479 (57.92%) | 17241235 (44.02%) |
| AA4 | 41900662 (96.7%) | 28303321 (67.55%) | 32286371 (77.05%) |
| AA5 | 38823886 (90.29%) | 25112249 (64.68%) | 27715470 (71.39%) |
| AM01 | 50534088 (83.12%) | 31426875 (62.19%) | 24703131 (48.88%) |
| AM02 | 73940456 (76.02%) | 35361389 (47.82%) | 39684951 (53.67%) |
| AM03 | 65535902 (90.25%) | 40070699 (61.14%) | 46793398 (71.4%) |
| AM04 | 64112638 (99.3%) | 54903406 (85.64%) | 55251327 (86.18%) |
| AM05 | 62744904 (99.14%) | 53371413 (85.06%) | 51926149 (82.76%) |
| U1 | 48191230 (95.35%) | 35539792 (73.75%) | 35567938 (73.81%) |
| U2 | 54546292 (98.36%) | 41113217 (75.37%) | 41629950 (76.32%) |
| U3 | 49627764 (97.49%) | 38493512 (77.56%) | 33536696 (67.58%) |
| U4 | 52186088 (98.82%) | 42063594 (80.6%) | 38170867 (73.14%) |
| U5 | 56641816 (96.66%) | 43044885 (75.99%) | 45704519 (80.69%) |

**Supplementary Table 7.** The most significantly differentially expressed genes involved in nitrogen metabolism. The comparison amino acid vs urea (AA/U), amino acid vs ammonia (AA/AM) and ammonia vs urea (AM/U)

| **Gene ID** | **Gene name** | **Gene description** | **Log2 FC(AA/U)** | **Log2 FC(AA/AM)** | **Log2 FC(AM/U)** |
| --- | --- | --- | --- | --- | --- |
| NPGAP_07905 | ureA | Urease gamma subunit | -3.5 | -2.7 | -0.8 |
| NPGAP_07920 | urtE | UreE urease accessory protein N-terminal domain | -3.9 | -3.5 | -0.3 |
| NPGAP_07935 | urtD | UreD urease accessory protein | -4.0 | -4.0 | 0.0 |
| NPGAP_08930 | PldB | Serine aminopeptidase S33 | -2.1 | -2.0 | -0.1 |
| NPGAP_10280 | ilvE | branched-chain amino acid aminotransferase | 3.6 | 1.7 | 1.9 |
| NPGAP_11490 | map | methionine aminopeptidase type I | -4.9 | -4.4 | -0.5 |
| NPGAP_11620 | MET17 | O-acetylhomoserine aminocarboxypropyltransferase/cysteine synthase | -9.3 | -9.0 | -0.4 |
| NPGAP_10365 | gltB | glutamine synthetase type I | -0.3 | 2.0 | -2.3 |
| NPGAP_09010 | ARO8 | Aminotransferase class I and II | -7.1 | -6.6 | -0.6 |
| NPGAP_12520 | GlyA | Serine hydroxymethyltransferase | -1.6 | -1.5 | -0.1 |
| NPGAP_12130 | mdh | malate dehydrogenase NAD-dependent | -1.1 | -1.5 | 0.5 |
| NPGAP_01555 | hisD | histidinol dehydrogenase | -4.3 | -5.0 | 0.8 |
| NPGAP_01550 | hisG | ATP phosphoribosyltransferase | -5.2 | -5.8 | 0.6 |
| NPGAP_01625 | dapE | succinyl-diaminopimelate desuccinylase | -2.1 | -2.6 | 0.6 |
| NPGAP_10420 | lysA | diaminopimelate decarboxylase | -0.6 | 0.8 | -1.5 |
| NPGAP_10345 | dapF | diaminopimelate epimerase | -0.6 | 0.8 | -1.5 |
| NPGAP_14640 | serC | phosphoserine transaminase | -5.5 | -5.9 | 0.4 |
| NPGAP_03580 | AnsA | L-asparaginase type II | -1.8 | -2.0 | 0.2 |
| NPGAP_08970 | MetC | Cys/Met metabolism PLP-dependent enzyme | -7.0 | -8.0 | 0.9 |
| NPGAP_08965 | CysK | Pyridoxal-phosphate dependent enzyme | -4.9 | -5.7 | 0.8 |
| NPGAP_00890 | AspA | Lyase | 1.3 | 1.0 | 0.3 |
| NPGAP_10340 | asnB | asparagine synthase (glutamine-hydrolyzing) | -4.5 | -4.1 | -0.5 |
| NPGAP_13390 | purF | amidophosphoribosyltransferase | 3.8 | 6.0 | -2.2 |
| NPGAP_10865 | speB | agmatinase | -6.0 | -6.2 | 0.2 |
| NPGAP_13775 | proB | glutamate 5-kinase | -1.8 | -1.7 | -0.1 |
| NPGAP_03285 | metK | methionine adenosyltransferase | -3.9 | -5.4 | 1.5 |
| NPGAP_06715 | asd | aspartate-semialdehyde dehydrogenase | -5.5 | -6.2 | 0.7 |
| NPGAP_14120 | ThrA | aspartate kinase | -1.4 | -2.1 | 0.7 |
| NPGAP_12045 | murE | UDP-N-acetylmuramyl-tripeptide synthetase | -5.9 | -5.1 | -0.8 |
| NPGAP_00995 | aroC | chorismate synthase | -1.7 | -1.5 | -0.2 |
| NPGAP_14370 | trpB | tryptophan synthase beta subunit | -4.1 | -3.8 | -0.3 |
| NPGAP_03140 | AroG1 | 3-deoxy-7-phosphoheptulonate synthase | -4.6 | -4.7 | 0.1 |
| NPGAP_05260 | FabG | short chain dehydrogenase | 1.9 | 1.7 | 0.2 |

**Supplementary Table 8**. Effects of different nitrogen sources on microbial protein production and enzyme activities involved in nitrogen metabolism and ammonia assimilation

| **Enzymes & MCP** | **Early phase** | | | **Mid Phase** | | | | **Late phase** | | | **SEM** | ***P*- value** | | |
| --- | --- | --- | --- | --- | --- | --- | --- | --- | --- | --- | --- | --- | --- | --- |
|  | **Urea** | **Ammonia** | **Amino acid** | **Urea** | **Ammonia** | | **Amino acid** | **Urea** | **Ammonia** | **Amino acid** |  | **Nitrogen sources** | **Phase** | **Nitrogen*Phase** |
| Urease (U/L) | 7.02^d^ | 1.77^f^ | 3.1^e^ | 20.45^b^ | 2.67^ef^ | 12.44^c^ | | 69.02^a^ | 8.01^d^ | 2.39^ef^ | 0.226 | <0.01 | <0.01 | <0.01 |
| GDH (U/min/mg) | 30.9^cd^ | 56.2^ab^ | 38.0^c^ | 42.02^bc^ | 65.75^a^ | 69.62^a^ | | 42.73^bc^ | 31.24^cd^ | 15.75^d^ | 6.062 | <0.05 | <0.01 | 0.01 |
| GOGAT (U/min/mg) | 7.23^bc^ | 10.17^ab^ | 9.87^ab^ | 8.08^bc^ | 12.64^a^ | 13.39^a^ | | 8.22^bc^ | 6.01^cd^ | 3.03^d^ | 0.727 | 0.26 | <0.01 | 0.01 |
| GS(U/min/mg) | 51.9^a^ | 7.24^b^ | 60.62^a^ | 6.69^b^ | 13.77^b^ | 12.3^c^ | | 6.69^b^ | 6.01^b^ | 3.72^b^ | 6.887 | <0.01 | <0.05 | <0.01 |
| MCP (µg/ml) | 79.31^c^ | 26.82^e^ | 16.99^e^ | 108.08^b^ | 48.98^d^ | 54.37^d^ | | 112.74^b^ | 113.74^b^ | 178.21^a^ | 3.282 | <0.01 | <0.01 | <0.01 |

Levels not connected by same letter are significantly different

Supplemntary Table 9. Correlation between DEGs and enzymes activity

|  | **ureA** | **urtE** | **urtD** | **PldB** | **ilvE** | **gltB** | **MET17** | **AroG1** | **map** | **serC** | **MetC** | **purF** | **asd** | **ThrA** | **murE** | **aroC** | **GDH** | **GOGAT** | **Urease** | **MCP** |
| --- | --- | --- | --- | --- | --- | --- | --- | --- | --- | --- | --- | --- | --- | --- | --- | --- | --- | --- | --- | --- |
| **ureA** |  |  |  |  |  |  |  |  |  |  |  |  |  |  |  |  |  |  |  |  |
| **urtE** | **0.860** |  |  |  |  |  |  |  |  |  |  |  |  |  |  |  |  |  |  |  |
|  | (0.003) |  |  |  |  |  |  |  |  |  |  |  |  |  |  |  |  |  |  |  |
| **urtD** | **0.528** | **0.422** |  |  |  |  |  |  |  |  |  |  |  |  |  |  |  |  |  |  |
|  | (0.144) | (0.258) |  |  |  |  |  |  |  |  |  |  |  |  |  |  |  |  |  |  |
| **PldB** | **0.839** | **0.643** | **0.775** |  |  |  |  |  |  |  |  |  |  |  |  |  |  |  |  |  |
|  | (0.005) | (0.062) | (0.014) |  |  |  |  |  |  |  |  |  |  |  |  |  |  |  |  |  |
| **ilvE** | **0.264** | **0.054** | **0.730** | **0.474** |  |  |  |  |  |  |  |  |  |  |  |  |  |  |  |  |
|  | 0.492 | 0.889 | (0.026) | (0.197) |  |  |  |  |  |  |  |  |  |  |  |  |  |  |  |  |
| **gltB** | **0.791** | **0.978** | **0.496** | **0.664** | **0.058** |  |  |  |  |  |  |  |  |  |  |  |  |  |  |  |
|  | (0.011) | (<0.001) | (0.175) | (0.051) | (0.882) |  |  |  |  |  |  |  |  |  |  |  |  |  |  |  |
| **MET17** | **0.694** | **0.884** | **0.490** | **0.695** | **-0.018** | **0.947** |  |  |  |  |  |  |  |  |  |  |  |  |  |  |
|  | (0.038) | (0.002) | (0.180) | (0.038) | (0.964) | (<0.001) |  |  |  |  |  |  |  |  |  |  |  |  |  |  |
| **AroG1** | **-0.604** | **-0.653** | **-0.696** | **-0.708** | **-0.427** | **-0.693** | **-0.705** |  |  |  |  |  |  |  |  |  |  |  |  |  |
|  | (0.085) | (0.057) | (0.037) | (0.033) | (0.251) | (0.038) | (0.034) |  |  |  |  |  |  |  |  |  |  |  |  |  |
| **map** | **0.842** | **0.839** | **0.767** | **0.925** | **0.348** | **0.884** | **0.891** | **-0.808** |  |  |  |  |  |  |  |  |  |  |  |  |
|  | (0.004) | (0.005) | (0.016) | (<0.001) | (0.359) | (0.002) | (0.001) | (0.008) |  |  |  |  |  |  |  |  |  |  |  |  |
| **serC** | **0.764** | **0.612** | **0.929** | **0.931** | **0.646** | **0.649** | **0.622** | **-0.697** | **0.898** |  |  |  |  |  |  |  |  |  |  |  |
|  | (0.016) | 0.080 | (0.001) | (0.001) | (0.060) | (0.059) | (0.074) | (0.037) | (0.001) |  |  |  |  |  |  |  |  |  |  |  |
| **MetC** | **0.834** | **0.888** | **0.713** | **0.874** | **0.296** | **0.930** | **0.935** | **-0.808** | **0.990** | **0.844** |  |  |  |  |  |  |  |  |  |  |
|  | (0.005) | (0.001) | (0.031) | (0.002) | (0.439) | (<0.001) | (<0.001) | (0.008) | (<0.001) | (0.004) |  |  |  |  |  |  |  |  |  |  |
| **purF** | **0.762** | **0.730** | **0.884** | **0.892** | **0.628** | **0.774** | **0.747** | **-0.844** | **0.942** | **0.945** | **0.925** |  |  |  |  |  |  |  |  |  |
|  | (0.017) | 0.026 | 0.002 | 0.001 | (0.070) | (0.014) | (0.021) | (0.004) | (<0.001) | (<0.001) | (<0.001) |  |  |  |  |  |  |  |  |  |
| **asd** | **0.876** | **0.902** | **0.719** | **0.834** | **0.435** | **0.904** | **0.846** | **-0.805** | **0.942** | **0.843** | **0.961** | **0.937** |  |  |  |  |  |  |  |  |
|  | (0.002) | (0.001) | 0.029 | 0.005 | (0.241) | (0.001) | (0.004) | (0.009) | (<0.001) | (0.004) | (<0.001) | (<0.001) |  |  |  |  |  |  |  |  |
| **ThrA** | **0.791** | **0.920** | **0.595** | **0.779** | **0.135** | **0.963** | **0.982** | **-0.762** | **0.945** | **0.729** | **0.979** | **0.840** | **0.930** |  |  |  |  |  |  |  |
|  | (0.011) | (<0.001) | (0.091) | (0.013) | (0.729) | (<0.001) | (<0.001) | (0.017) | (<0.001) | (0.026) | (<0.001) | (0.005) | (<0.001) |  |  |  |  |  |  |  |
| **murE** | **0.894** | **0.882** | **0.770** | **0.877** | **0.419** | **0.890** | **0.845** | **-0.796** | **0.964** | **0.887** | **0.969** | **0.942** | **0.988** | **0.930** |  |  |  |  |  |  |
|  | (0.001) | (0.002) | (0.015) | (0.002) | (0.262) | (0.001) | (0.004) | (0.010) | (<0.001) | (0.001) | (<0.001) | (<0.001) | (<0.001) | (<0.001) |  |  |  |  |  |  |
| **aroC** | **0.553** | **0.772** | **0.443** | **0.630** | **-0.107** | **0.867** | **0.977** | **-0.641** | **0.826** | **0.544** | **0.868** | **0.661** | **0.734** | **0.928** | **0.742** |  |  |  |  |  |
|  | (0.122) | (0.015) | (0.233) | (0.069) | (0.783) | (0.002) | (<0.001) | (0.063) | (0.006) | (0.130) | (0.002) | (0.052) | (0.024) | (<0.001) | (0.022) |  |  |  |  |  |
| **GDH** | **-0.367** | **-0.640** | **-0.382** | **-0.238** | **0.163** | **-0.707** | **-0.614** | **0.260** | **-0.513** | **-0.395** | **-0.539** | **-0.403** | **-0.474** | **-0.589** | **-0.509** | **-0.590** |  |  |  |  |
|  | (0.332) | (0.063) | (0.311) | (0.538) | (0.675) | (0.033) | (0.079) | (0.499) | (0.158) | (0.293) | (0.134) | (0.282) | (0.198) | (0.095) | (0.161) | (0.094) |  |  |  |  |
| **GOGAT** | **-0.367** | **-0.640** | **-0.382** | **-0.238** | **0.163** | **-0.707** | **-0.614** | **0.260** | **-0.513** | **-0.395** | **-0.539** | **-0.403** | **-0.474** | **-0.589** | **-0.510** | **-0.591** | **1.000** |  |  |  |
|  | (0.332) | (0.063) | (0.311) | (0.538) | (0.674) | (0.033) | (0.079) | (0.499) | (0.158) | (0.293) | (0.134) | (0.282) | (0.198) | (0.095) | (0.161) | (0.094) | (<0.001) |  |  |  |
| **Urease** | **0.246** | **0.597** | **-0.294** | **0.010** | **-0.709** | **0.616** | **0.658** | **-0.115** | **0.280** | **-0.135** | **0.375** | **0.015** | **0.264** | **0.540** | **0.236** | **0.683** | **-0.526** | **-0.526** |  |  |
|  | (0.524) | (0.090) | (0.443) | (0.979) | (0.033) | (0.077) | (0.054) | (0.768) | (0.466) | (0.730) | (0.320) | (0.969) | (0.492) | (0.134) | (0.541) | (0.042) | (0.146) | (0.146) |  |  |
| **MCP** | **0.507** | **0.831** | **0.215** | **0.354** | **-0.349** | **0.881** | **0.879** | **-0.525** | **0.651** | **0.320** | **0.716** | **0.456** | **0.620** | **0.818** | **0.617** | **0.865** | **-0.787** | **-0.788** | **0.844** |  |
|  | (0.163) | (0.005) | (0.578) | (0.349) | (0.357) | (0.002) | (0.002) | (0.146) | (0.058) | (0.401) | (0.030) | (0.217) | (0.075) | (0.007) | (0.076) | (0.003) | (0.012) | (0.012) | (0.004) |  |
| **GS** | **-0.448** | **-0.739** | **-0.152** | **-0.261** | **0.459** | **-0.774** | **-0.780** | **0.440** | **-0.540** | **-0.227** | **-0.600** | **-0.321** | **-0.503** | **-0.716** | **-0.526** | **-0.778** | **0.772** | **0.772** | **-0.824** | **-0.963** |
|  | (0.227) | (0.023) | (0.696) | (0.498) | (0.214) | (0.014) | (0.013) | (0.236) | (0.134) | (0.557) | (0.088) | (0.399) | (0.168) | (0.030) | (0.146) | (0.014) | (0.015) | (0.015) | (0.006) | (<0.001) |

**Note:** The values in the bracket represent P-value and the value marked in yellow represent significantly different.

**Supplementary Table 10. Primers used to see the expression of genes in qRT-PCR analysis**

| **Target gene** | **Forward primer (5’ to 3’)** | **Reverse primer(5’ to 3’)** | **Product size(bp)** |
| --- | --- | --- | --- |
| ilvE | GCTGTATTTGAGGGTATTCGTG | CAGACCATAGCATCGGCATT | 266 |
| map | TACTGTGCTGAAGGCTGTTTGTC | GCAGATGTGCCCAGGAAGG | 325 |
| Met17 | AGTCTACAATCACTCCTCCCAAT | CATCTACGGCGGCTCCTATA | 344 |
| serC | AGGAGGCTCAGCGTTCTTTC | GCACTCTGTAATTGGTTGGGATAT | 150 |
| MetC | AATGTCCGCCTATGTACTTGGT | ATGCTGGCTTTGCCTTCG | 405 |
| purF | CTGGTGCTAATAAGGTTTACTTCG | CAGATTTCCTCGTTGGTTCG | 124 |
| asd | GGTGCCTTCTGACCTGTCTGT | TTGGATGGCGTGGTATGG | 115 |
| ThrA | GCTGAGTGCTGTGAGATATGGAC | CCCCTTTGGGTTCTTGGT | 213 |
| 16S rRNA | GAACTTAGGGACGCTTTGTGG | CGTGTCGTGAGATGTTGGGT | 234 |
